# Impact of Synonymous Genome Recoding on the HIV Life Cycle

**DOI:** 10.3389/fmicb.2021.606087

**Published:** 2021-03-16

**Authors:** Ana Jordan-Paiz, Sandra Franco, Miguel Angel Martínez

**Affiliations:** IrsiCaixa, Hospital Universitari Germans Trias i Pujol, Universitat Autònoma de Barcelona (UAB), Badalona, Spain

**Keywords:** HIV-1, synonymous, mutations, virus, phenotype

## Abstract

Synonymous mutations within protein coding regions introduce changes in DNA or messenger (m) RNA, without mutating the encoded proteins. Synonymous recoding of virus genomes has facilitated the identification of previously unknown virus biological features. Moreover, large-scale synonymous recoding of the genome of human immunodeficiency virus type 1 (HIV-1) has elucidated new antiviral mechanisms within the innate immune response, and has improved our knowledge of new functional virus genome structures, the relevance of codon usage for the temporal regulation of viral gene expression, and HIV-1 mutational robustness and adaptability. Continuous improvements in our understanding of the impacts of synonymous substitutions on virus phenotype – coupled with the decreased cost of chemically synthesizing DNA and improved methods for assembling DNA fragments – have enhanced our ability to identify potential HIV-1 and host factors and other aspects involved in the infection process. In this review, we address how silent mutagenesis impacts HIV-1 phenotype and replication capacity. We also discuss the general potential of synonymous recoding of the HIV-1 genome to elucidate unknown aspects of the virus life cycle, and to identify new therapeutic targets.

## Introduction

Despite the relatively recent introduction of HIV-1 within the human population, this virus has already exhibited enormous diversification. This high genetic diversity results from its fast replication cycle, with the generation of about 10^10^ virions daily in an infected individual, coupled with its high mutation rate of approximately 3 × 10^–5^ per nucleotide base per replication cycle, and the recombinogenic properties of its reverse transcriptase (RT) (Coffin and Swanstrom, 2013). The HIV-1 RNA genome comprises an above-average proportion of adenine (A) nucleotides, while being extremely poor in cytosine (C) (van der Kuyl and Berkhout, 2012). Interestingly, despite the high variability of the HIV-1 genome, its base composition is surprisingly stable over time, varying by <1% per base per isolate regardless of whether it originates from the early or later years of the epidemic (van der Kuyl and Berkhout, 2012). This stability of the peculiar base composition of the HIV-1 genome strongly impacts its synonymous codon and codon pair usage, as well as dinucleotide frequencies.

All organisms share the same genetic code, in which four different nucleotides generate codon triplets – such that 4^3^, or 64, different codons are possible. These 64 codons encode 20 different amino acids and 3 translation stop codons. Of these 20 amino acids, 18 are encoded by more than one synonymous codon, and only methionine and tryptophan are encoded by a unique triplet codon. The ratios of synonymous codons are highly non-random, i.e., some synonymous codons appear more frequent than others (Grantham et al., 1980), a phenomenon termed codon usage bias. Codon usage differs among different species, strongly suggesting that codon usage is an adaptive trait affected by selective pressure and random drift. Another bias that can be observed in organism genomes is that codon pair frequencies are not random, which is termed codon-pair bias. The frequencies of codon-pairs can be different from what would be expected based on the individual codon usage bias of a given genome, as reviewed by Alexaki et al. (2019). Indeed, codon-pair bias have been also described in other organisms, including HIV-1 (Martrus et al., 2013).

In addition to the translation and abundance of isoaccepting tRNAs, synonymous codon mutations can impact many other molecular phenotypes, including transcription modifications (Zhou et al., 2016; Findlay et al., 2018), translation initiation (Kudla et al., 2009; Goodman et al., 2013; Stergachis et al., 2013), translation elongation (Sorensen et al., 1989; Boel et al., 2016), translation accuracy (Akashi, 1994; Drummond and Wilke, 2008), RNA stability (Presnyak et al., 2015), RNA structure and folding (Shabalina et al., 2006; Kudla et al., 2009), RNA splicing (Pagani et al., 2005; Takata et al., 2018), RNA toxicity (Mittal et al., 2018), cotranslational folding (Pechmann and Frydman, 2013), chromatin organization (Warnecke et al., 2009), enhancer functions (Lin et al., 2011; Birnbaum et al., 2012), and microRNA targeting (Brest et al., 2011; Birnbaum et al., 2012). These impacts of synonymous mutations on cell phenotype further indicate that the distribution of synonymous substitutions throughout genes and genomes is neither random nor neutral, and is thus subjected to selective forces. As mentioned above, HIV-1 is a good example since, although the genome is highly variable, the genomic base composition has been tremendously stable over time.

**FIGURE 1:**
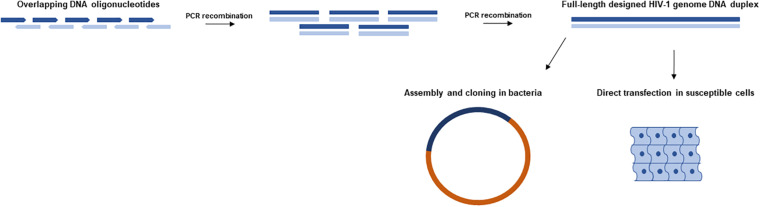
Synthetic HIV-1 DNA oligonucleotide assembly and cell transfection. Overlapping oligonucleotides encoding a full-length HIV-1 genome DNA duplex are successively assembled via PCR or isothermal amplification. The designed HIV-1 DNA duplex can then be directly transfected into susceptible cells (e.g., MT-4 and PBMCs) to yield infectious virus (Fujita et al., 2013). Alternatively, synthetic HIV-1 DNA can be cloned in bacteria or other vectors for further manipulation.

**FIGURE 2:**
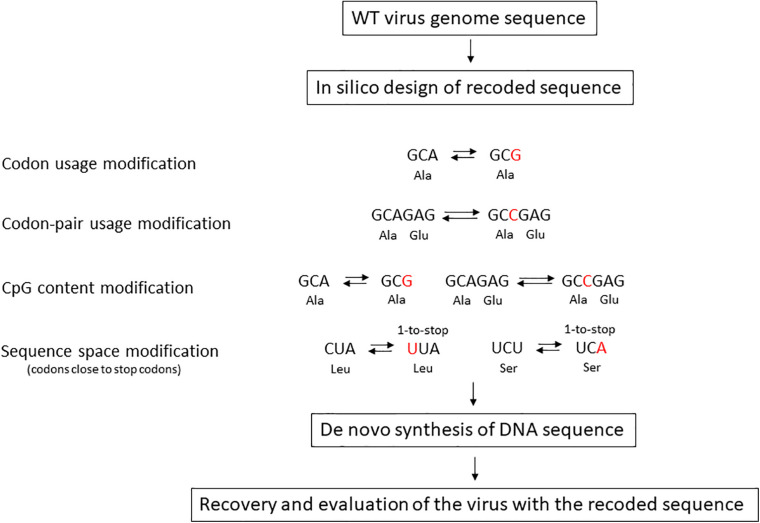
Methods for genome synonymous recoding. Four main strategies have been used to synonymously recode virus genome sequences: codon usage modification, codon-pair usage modification, CpG content modification, and modification of codons that can generate stop mutations after a single nucleotide substitution.

Forty years ago, the invention of PCR and the chemical synthesis of DNA oligonucleotides opened the door to synthetic genomics. Nowadays, DNA chemical synthesis enables the synthesis of 200-nucleotide-long oligonucleotides (Hughes and Ellington, 2017). Overlapping single-stranded DNA oligonucleotides of 100–200 nucleotides in length can easily be assembled by PCR or isothermal amplification to generate DNA fragments of 1,000–2,000 base pairs (bps) (Figure 1). Conventional cloning methods can be applied to clone these synthetic DNA fragments into a bacterial plasmid vector, and individual clones can be isolated and sequenced by Sanger sequencing. Virus genome recoding is a recent tool that is enabling us to elucidate fundamental aspects of virus biology (Figure 2). Synthetic recoding can also help us to develop better therapeutic tools, such as new synthetic vaccines and virus-based gene therapy vectors. Next, we will discuss how synthetic HIV-1 synonymous genome recoding (Table 1) is uncovering HIV-1 biology, and opening the door to new therapeutic opportunities. Remarkably relevant is how HIV-1 synonymous genome recoding has allowed the description of previous unknown functions of cell factors involved in the innate immune response (Li et al., 2012; Takata et al., 2017).

**TABLE 1:** Examples of HIV-1/SIV phenotype modification by genome synonymous substitutions.

**Targeted Gene**	**Recoding method**	**Implicated host factor**	**Resulting phenotype**	**References**
*gag*	Codon usage	Innate response (SLFN11)	Translation inhibition (lethality)	Li et al., 2012
*gag, pol*	Codon-pair usage	ND	Translation inhibition (attenuation, lethality)	Martrus et al., 2013
*env*	Codon usage	Innate response (Type 1 interferon)	Transcription inhibition (attenuation)	Vabret et al., 2014
*gag, pol, env*	CpG content	Innate response (ZAP)	Transcription inhibition (attenuation, lethality)	Takata et al., 2017
*pol*	Codon-pair usage	None	Sequence space modification (none)	Nevot et al., 2018
*gag, pol, env*	CpG content	mRNA splicing	Suppression of splice sites (attenuation, lethality)	Takata et al., 2018
*env*	Codon usage	mRNA splicing	Suppression of essential mRNA structures (attenuation, lethality)	Jordan-Paiz et al., 2020

*ND, not determined.*

## Synonymous Substitutions and HIV-1 Replication Capacity

**FIGURE 3:**
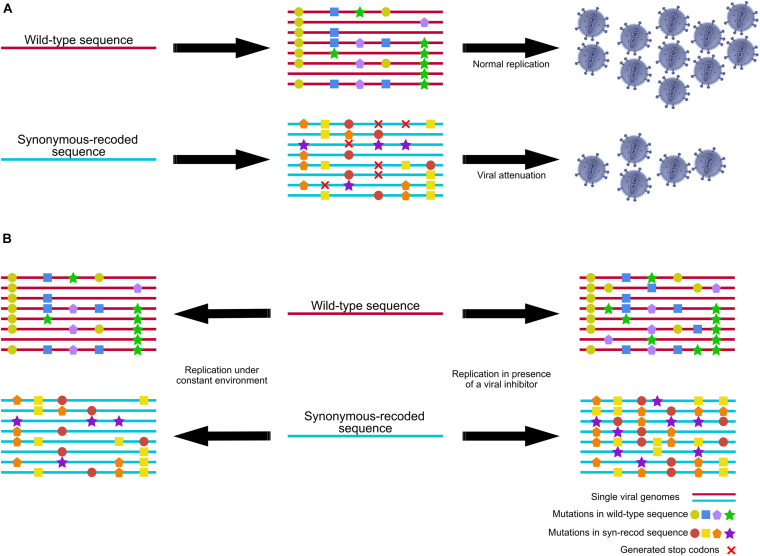
Viral population diversity and evolvability of a wild-type sequence and a synonymously recoded sequence. **(A)** Synonymous recodification of a viral sequence leads to a different and limited mutant spectra which may result in the generation of stop codons. The generation of stop codons in the mutant spectra results in virus attenuation (Lauring et al., 2012; Moratorio et al., 2017). **(B)** Replication of a wild-type (WT) virus and a synonymous-recoded virus under a constant environment may lead to different mutant spectra, but not necessarily to generation of stop codons or viral attenuation. Under the presence of a viral inhibitor, the number of mutations found in the synonymous-recoded virus is higher than those generated in the WT virus. Moreover, these mutations differ from both viral mutant spectra, indicating that the sequence space influences the development of inhibitor resistances (Nevot et al., 2018).

A well-known and common application of synonymous nucleotide recoding is synonymous codon optimization to increase protein expression in various systems (Mauro and Chappell, 2014). On the other hand, an interesting and less known application of synonymous nucleotide recoding is to synonymously deoptimize codon usage, codon-pair usage, or dinucleotide frequencies to reduce protein expression and attenuate virus replication capacity, which has been described for several RNA viruses (Figure 3; Martínez et al., 2016, 2019). Recoding viral genomes through numerous synonymous but suboptimal substitutions represents a new source of live attenuated vaccine candidates. In pioneer research with poliovirus, the introduction of 542 synonymous substitutions among the 2,555 nucleotides of the virus capsid region reduced the virus replication capacity by up to 98% in HeLa cells (Burns et al., 2006). A similar approach involving synonymous deoptimization of the poliovirus capsid coding region generated a virus that exhibited a neuro-attenuated phenotype in transgenic mice (Mueller et al., 2006). Large-scale synonymous codon usage recoding has been used to generate prototypes of live attenuated vaccines for several RNA viruses, including poliovirus (Burns et al., 2006; Mueller et al., 2006), influenza virus (Mueller et al., 2010; Yang et al., 2013), respiratory syncytial virus (RSV) (Nouën et al., 2014), vesicular stomatitis virus (Wang et al., 2015), porcine reproductive and respiratory syndrome virus (Ni et al., 2014), dengue virus (Shen et al., 2015), zika virus (Li et al., 2018), echovirus 7 (Fros et al., 2017), foot and mouth disease virus (Diaz-San Segundo et al., 2021), and the plant cucumber mosaic virus (Mochizuki et al., 2018); arboviruses, such as Chikungunya virus (Nougairede et al., 2013) and tick-borne encephalitis virus (de Fabritus et al., 2015); and DNA viruses, such as Marek’s disease herpesvirus (Conrad et al., 2018; Eschke et al., 2018). Clinical trials have been performed using codon-deoptimized type 2 poliovirus. These trials found the vaccine candidate to be safe and immunogenic in infants and toddlers (Van Damme et al., 2019; Konopka-Anstadt et al., 2020; De Coster et al., 2021; Sáez-Llorens et al., 2021).

The HIV-1 genome has been also synonymously deoptimized (Martrus et al., 2013; Klaver et al., 2017). In one study, up to 118 substitutions were introduced in the 1,508-nucleotide structural Gag-coding region, generating viruses with lower replicative capacities in an established MT-4 cell line and in peripheral blood mononuclear cells (PBMCs) from uninfected donors (Martrus et al., 2013). The replication capacity of these recoded variants was reduced up to 39 and 85% in MT-4 cells and PBMCs, respectively (Martrus et al., 2013). Similarly, the introduction of 41 substitutions in protease coding region also generated a virus with reduced replication. To test the phenotypic stability of these protease and *gag* variants, the viruses were serially propagated in MT-4 cells, revealing that all deoptimized viruses recovered wild-type (WT) replication capacity after 60 days of cell culture propagation (Martrus et al., 2013). Individual virus clones were obtained after cell culture passages, and sequencing revealed that several deoptimizing synonymous substitutions had reverted to WT; many additional synonymous and non-synonymous mutations were also detected. The clinical development of an attenuated HIV-1 vaccine is thus improbable. However, these experiments confirm the rapid evolution of an RNA virus, and the necessity of rigorous experiments to evaluate the stability of all candidates for new attenuated virus vaccines based on genome synonymous recoding.

In another lentivirus, simian immunodeficiency virus (SIV), the introduction of 169 synonymous nucleotide optimizing mutations in *gag* and *pol* yielded a virus with a 100-fold decrease of its replication capacity (Vabret et al., 2014). Interestingly, the recoded virus exhibited a reduced ability to stimulate type I interferon, which may have attenuated its pathogenic potential. Analogously, synonymous deoptimization of the *Streptococcus pneumoniae* pneumolysin gene with underrepresented codon pairs resulted in an attenuated phenotype in a mouse pulmonary infection model, which was associated with a markedly reduced inflammatory response in the lungs (Coleman et al., 2011).

Reports have also described *cis*-acting functions in the HIV-1 coding sequence for viral gene expression. Synonymous adaptive mutations in the HIV-1 3’ *pol* gene result in parallel increases or decreases in the expression levels of late viral proteins and in viral replication capacity (Nomaguchi et al., 2014). These findings suggest that viral fitness is altered through nucleotide-dependent modulation of the expression pattern of viral mRNAs. A global silent mutagenesis experiment was performed to identify new *cis*-acting RNA elements in the HIV-1 genome that are important for virus replication (Takata et al., 2018). Sixteen mutant proviruses were designed and synthesized, which contained clusters of ∼50 to ∼200 synonymous mutations spanning nearly the entire HIV-1 protein coding sequence. These mutant viruses were analyzed and categorized into three phenotypic groups: (1) mutants exhibiting near WT replication, (2) mutants exhibiting replication defects accompanied by perturbed RNA splicing, and (3) mutants exhibiting replication defects without obvious splicing perturbation (Takata et al., 2017). Mutants of the second group generally contained point mutations that reduced proximal splice site utilization. Mapping the changes responsible for splicing perturbations in these viruses revealed several RNA sequences that apparently suppressed the use of cryptic or canonical splice sites. These findings indicated complex negative regulation of HIV-1 splicing via RNA elements in various regions of the HIV-1 genome, which maintained a balance between splicing and viral replication (Takata et al., 2018). Overall, these experiments demonstrated that synonymous HIV-1 recoding may provide insights into uncharacterized elements in the HIV-1 genome that determine the fate and splicing of HIV-1 RNA and thus the ability of HIV-1 to replicate.

## Synonymous Sequence Space

One unknown aspect of the genetic architecture of RNA viruses, including HIV-1, is how codon choice influences population diversity and evolvability. Early comparisons of the nucleotide sequences of homologous genes revealed higher numbers of synonymous substitutions compared to non-synonymous mutations, promoting an initial assumption that synonymous mutations were selectively neutral. This postulation contributed to the foundations of the neutral theory of molecular evolution, in which organisms evolve mainly through the random drift of genomes carrying neutral or quasi-neutral mutations (King and Jukes, 1969; Kimura, 1983). However, although genome random drift may play an important role in molecular evolution, the currently available evidence indicates that synonymous mutations are not neutral (Martínez et al., 2019; Domingo, 2020).

Synonymous substitutions can determine the evolutionary trajectory of a genome. Different codons that encode the same amino acid can have different evolutionary potential in terms of the amino acids that they can access through a point mutation, which can determine their likelihood of reaching beneficial mutations that facilitate adaptation (Lauring et al., 2013). Research with polio, coxsackie B3, and influenza A viruses has revealed that synonymous recoding of the virus genome can change its starting position in sequence space and limit its access to mutational neighborhoods (Lauring et al., 2012; Moratorio et al., 2017), potentially resulting in virus attenuation. Mutational neighborhoods refer to a network of variants organized in sequence space around a single master sequence (Domingo, 2020). In the cases of coxsackie B3 and influenza A viruses, leucine and serine codons were recoded to favor the possibility of nonsense mutations resulting in stop codons. The virus variants were attenuated *in vivo* and exhibited increased numbers of stop codons. These findings suggested the possibility of changing a virus’ starting position in sequence space, and redirecting it toward detrimental mutational neighborhoods to generate self-limiting vaccine strains (Moratorio et al., 2017). Similarly, a synonymously recoded poliovirus exhibited unique mutant spectra, showing significantly different distributions of polymorphic amino acid substitutions in the capsid (Lauring et al., 2012). This recoded virus exhibited normal replication capacity in tissue culture, but displayed an attenuated phenotype in an animal model of infection, demonstrating the importance of mutant neighborhoods in determining viral pathogenesis.

To explore whether the synonymous sequence space influences the development of HIV-1 protease inhibitor (PI) resistance, WT HIV-1 was compared to a variant carrying a protease gene with 38 synonymous mutations (13% of the protease sequence) (Nevot et al., 2018). The 38 synonymous substitutions were scattered throughout the protease coding region, and were selected to improve protease gene codon-pair bias without modifying the codon bias or folding free energy (Martrus et al., 2013). Importantly, replication in MT-4 cells or PBMCs was indistinguishable between the recoded variant and the WT virus. Similar to the studies performed with poliovirus and coxsackie virus, this investigation was designed to explore how HIV-1 evolvability was influenced by the natural position in sequence space. In contrast to previous work, this study explored how synonymous substitutions affected the specific selection pressure targeting a precise HIV-1 gene. Interestingly, upon PI treatment, the recoded virus displayed different patterns of resistance mutations, demonstrating that sequence space position affects evolvability. However, although the WT and recoded proteases occupied different sequence spaces, they showed similar levels of development of phenotypic resistance to PIs – i.e., the recoded and WT virus exhibited the same robustness to overcome a specific selective pressure (Nevot et al., 2018). Interestingly, the recoded virus showed significantly higher population diversity in the recoded and targeted gene following propagation in both the absence and presence of PIs. It is tempting to speculate that the recoded virus was subjected to greater pressure to change or revert to a WT-synonymous background. As discussed above, positioning a virus in a detrimental sequence space to reduce its capacity to produce fit progeny may be a new strategy for attenuated poliovirus vaccine development (Lauring et al., 2012). However, the research involving the HIV-1 protease (Nevot et al., 2018) suggests that this approach must be cautiously developed, with careful testing of the long-term stability of synonymously recoded individual candidate viruses.

## Synonymous Substitutions and HIV-1 RNA Secondary Structure

RNA virus genomes contain multiple functional RNA elements that are required for translation or RNA replication. Synonymous genome recoding has been exploited to identify specific RNA structures required for virus replication. Recoding does not affect virus growth unless it destroys the sequence/structure of a functional RNA element (Song et al., 2012). SHAPE experiments have revealed that in HIV-1 RNA, individual nucleotides exhibit widely divergent tendencies to be base-paired (Wilkinson et al., 2008; Watts et al., 2009). HIV-1 RNA secondary structures are conserved between strains, and thus might have a function in HIV-1 replication. As previously discussed, synonymous recoding of the HIV-1 genome has elucidated the presence of unknown RNA *cis*-regulatory elements that influence balanced splicing and viral replication (Takata et al., 2018). Using dimethyl sulfate mutational profiling and sequencing (DMS-MaPseq) to investigate the HIV-1 RNA structure in cells has revealed that the same RNA sequence can assume alternative conformations (Tomezsko et al., 2020). These findings have revealed heterogeneous regions of RNA structure across the entire HIV-1 genome, as well as alternative conformations at critical splice sites that influence the ratio of transcript isoforms. Overall, these results strongly suggest that HIV-1 RNA conformation regulates splice-site use and viral gene expression (Tomezsko et al., 2020).

In SIV and HIV-1, the expression of Env depends on the nature of the codons used (Shin et al., 2015). One interesting hypothesis is that in different families of persistent viruses, codon usage is skewed in a distinctive manner to enable temporal regulation of late-expressed structural gene products, which is the case for HIV-1 Env. Temporal regulation of the lentiviral Env protein ensures its production late in the lytic cycle of these persisting viruses. Notably, expression of these late gene products is typically induced by viral transducers produced earlier in the viral replication cycle. One example of such a transducer is the HIV-1/SIV protein Rev. A nuclear localization signal encoded in the *rev* gene enables localization of the Rev protein to the nucleus, where it participates in the export of unspliced and incompletely spliced mRNAs. In the absence of Rev, mRNAs of the late (structural) HIV-1/SIV genes (e.g., Env protein) are retained in the nucleus, preventing their translation (Karn and Stoltzfus, 2012).

Env exhibits unusual codon usage that differs from that of the host cell. It was recently demonstrated that Rev induction of Env protein expression is dependent on this biased codon usage (Shin et al., 2015). To determine whether codon usage affected HIV-1 Env protein expression and virus viability, the codons AGG, GAG, CCU, ACU, CUC, and GGG of the HIV-1 *env* gene were substituted with the synonymous codons CGU, GAA, CCG, ACG, UUA, and GGA, respectively (Jordan-Paiz et al., 2020). This approach revealed that synonymous recoding of the Env protein gp120 coding region did not significantly affect virus replication capacity, despite the introduction of 15 new CpG dinucleotides (see in the next section the relevance of CpGs). In contrast, changing a single codon (AGG to CGU) within the gp41 coding region (HXB2 *env* position 2,125–2,127), which is located in the intronic splicing silencer (ISS), completely abolished virus replication and Env expression (Jordan-Paiz et al., 2020). Computational analyses of this mutant revealed severe disruption of the ISS RNA secondary structure. Moreover, a variant that restored the ISS secondary RNA structure also re-established Env production and virus viability. These findings indicate that external ISS loop disruption strongly affected HIV-1 replication and Env translation – again highlighting the relevance of synonymous recoding in maintaining biologically relevant RNA structures.

## HIV-1 Dinucleotide Frequencies and Innate Immune Response

As previously discussed, lentiviral RNA genomes (e.g., HIV-1 and SIV) exhibit a strong bias in their nucleotide composition, with high frequencies of A and low content of C (van der Kuyl and Berkhout, 2012). In accordance with the nucleotide composition, a biased dinucleotide frequency is also observed in HIV-1, with the most frequent occurrence of the dinucleotide ApA and a lower-than-expected proportion of CpG. Reduced CpG frequencies are also observed in many other RNA viruses, and in most vertebrate genomes. The low CpG abundance in vertebrate genomes can be explained by the methylation/deamination process that promotes the mutation of CpG to TpG/CpA (Karlin and Mrázek, 1997). In contrast to HIV-1, although many RNA viruses mimic the CpG suppression of their vertebrate hosts (Simmonds et al., 2013), this phenomenon cannot be explained by methylation since they lack a DNA intermediate. Thus, the explanation for the low CpG abundance in RNA viruses remains controversial.

Studies of several RNA viruses, including HIV-1, have revealed that increases of CpG in the virus genome negatively impact their replication capacity (Burns et al., 2009; Atkinson et al., 2014; Tulloch et al., 2014; Gaunt et al., 2016; Antzin-Anduetza et al., 2017; Takata et al., 2017). In HIV-1-infected individuals, the *in vivo* generation of *de novo* CpG sites carries twice the fitness cost of mutations that do not generate CpG sites (Theys et al., 2018). These findings raise questions regarding why CpG sites are suppressed in the HIV-1 genome, and why an increase of this dinucleotide carries a fitness cost.

**FIGURE 4:**
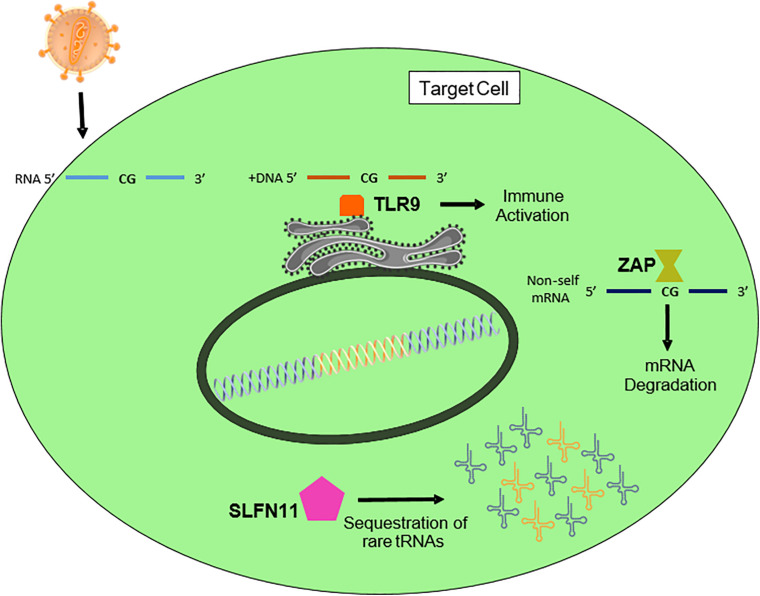
Factors of the innate immune system that might affect the nucleotide composition of the HIV-1 genome. SLFN11 sequesters tRNAs in a codon-dependent manner. Toll-like receptor 9 recognizes unmethylated CpGs in DNA, and activates the immune cascade (Pandey et al., 2015). Finally, ZAP recognizes CpG-rich non-self RNAs and induces their degradation.

Toll-like receptor 9 (TLR9) recognizes bacterial and viral DNA that is rich in unmethylated CpG DNA (Figure 4; Pandey et al., 2015). TLR9 is highly expressed in plasmacytoid dendritic cells. Since the replication of HIV-1 in plasmacytoid dendritic cells is not fully demonstrated, the role of TLR9 in HIV-1 CpG suppression is also unclear. Recently, Takata and colleagues generated several CpG-rich HIV-1 variants through the synthetic random synonymous mutagenesis of different HIV-1 regions (Takata et al., 2017). As previously described (Atkinson et al., 2014; Gaunt et al., 2016; Antzin-Anduetza et al., 2017), they found that variants containing larger numbers of CpG sites had significantly lower replication capacity. By targeting different cellular proteins implicated in mRNA degradation, they revealed that knockdown of a zinc-finger antiviral protein (ZAP) restored the normal replication capacity of CpG-rich HIV-1 variants (Figure 4). ZAP is a factor involved in the innate immune response, which was first described as an inhibitor of viral RNA production (Gao et al., 2002). ZAP specifically binds viral mRNAs (Guo et al., 2004) and prevents their cytoplasmic accumulation by recruiting and promoting degradation of the RNA processing exosome (Guo et al., 2007) – thereby promoting specific loss of cytoplasmic viral mRNA without affecting nuclear mRNA. While previous studies could not determine how ZAP targets viral mRNA (Guo et al., 2004), Takata and colleagues demonstrated that ZAP showed specificity for CpG-rich HIV-1 mRNA exonic regions. These authors suggested that RNA virus evolution may have favored low CpG content to avoid selective inhibition by ZAP. Examination of another RNA virus has corroborated the relationship between CpG frequency, ZAP, and virus replication capacity (Odon et al., 2019).

Since the demonstration that ZAP induces the degradation of viral CpG-rich mRNA, several studies have focused on the mechanism underlying this binding and inhibition. As previously described, ZAP recruits the RNA processing exosome (Guo et al., 2007) and this complex degrades CpG-rich viral mRNAs. However, the regulation of ZAP’s antiviral activity is not completely understood, as other cellular cofactors might be involved in its activation. The E3 ubiquitin ligase TRIM25 reportedly enhances ZAP and is required for its activity (Li et al., 2017; Zheng et al., 2017). ZAP interacts with several components of the RNA exosome complex, suggesting that TRIM25 may not be the only cofactor that mediates ZAP activity. Indeed, it was recently demonstrated that KHNYN, a newly described cytoplasmic human protein, is also essential for ZAP activity against foreign CpG-rich mRNAs (Ficarelli et al., 2019b). This study revealed how KHNYN interacted with ZAP, and that its overexpression dramatically reduced the infectivity capacity of a synonymous recoded CpG-rich HIV-1 variant. Moreover, KHNYN inhibition enabled this CpG-rich variant to reach WT replication levels.

The N-terminal domain of ZAP includes four CCCH-type zinc-finger motifs that together form the ZAP RNA-binding domain (RBD), and are responsible for targeting viral mRNA (Chen et al., 2012). A recent paper describes the RBD structure, and how it selectively binds CpG-rich mRNA sequences (Meagher et al., 2019). Structural analysis of ZAP revealed a pocket on the protein surface, which only binds CpG dinucleotides (Meagher et al., 2019). This ZAP structure may explain how ZAP avoids low-CpG host mRNA and recognizes foreign mRNAs. Importantly, another recent publication describes the molecular mechanism through which ZAP only detects CpG sites in a single-stranded form (Luo et al., 2020). The four zinc-finger motifs of ZAP form a specific architecture that enables extensive interactions with RNA. They further revealed that an RNA containing several ZAP-binding sites can be recognized by multiple ZAP molecules, which likely enhances the activation of the exosome complex. The authors hypothesize that a single CpG dinucleotide cannot bind ZAP and activate the exosome complex, but rather must be single-stranded and surrounded by other CpG sequences that can bind several ZAP molecules, potentially exerting a synergistic effect.

Despite recent descriptions of the ZAP structure and molecular mechanism, there remains some controversy surrounding how CpG-rich mRNAs are targeted. Investigations of the genomes of different primate lentiviruses do not reveal any correlation between the number of CpG sites and the sensitivity to ZAP. As previously noted, the HIV-1 genome contains a very low proportion of CpG sites. In contrast, HIV-2 shows less CpG suppression. However, although the CpG content is higher in HIV-2 than in HIV-1, no ZAP inhibition is observed in HIV-2 (Kmiec et al., 2020). This phenomenon has also been observed for another lentivirus, SIVmac, which exhibits a higher CpG content but significantly lower ZAP inhibition compared to HIV-1. This raises the question of why ZAP can inhibit HIV-1, but not other related lentiviruses.

It is hypothesized that a region in the genome, termed ZAPsen, may confer ZAP sensitivity. In HIV-1, this ZAPsen region is described as located in the 5’ region of the *env* gene (from position 6,239–6,947 of the HIV-1 HXB2 reference genome) (Kmiec et al., 2020). And increase of synonymous CpG sites in ZAPsen confers higher ZAP sensitivity and lower virus replication capacity (Ficarelli et al., 2019a; Kmiec et al., 2020). However, this is not the only factor that affects replication. ZAP sensitivity is also increased by the introduction of other ZAPsen mutations that alter the RNA secondary structure, without modifying the number of CpGs. Thus, ZAP may detect the ZAPsen region based on either the number of CpG sites, or changes in the RNA secondary structure. ZAP shows greater affinity to ZAPsen when higher amounts of CpGs are introduced, but other factors may also explain why CpG suppression is observed along the whole HIV-1 genome. Accordingly, synonymous mutations that increase the number of CpGs in other HIV-1 regions also lead to reduced replication capacity (Ficarelli et al., 2019a). These variants were not inhibited by ZAP, but rather through other ZAP-independent mechanisms, mainly related to pre-mRNA splicing. As described in a previous section, synonymous mutations can also alter splicing (Takata et al., 2018). In this case, synonymous CpGs were responsible for aberrant mRNA splicing and reduced viral fitness.

As discussed above, CpG suppression in HIV-1 might be partly explained by the actions of the innate response factor ZAP. However, other mechanisms may also contribute, including mRNA splicing and mRNA secondary structure. The exact mechanism of ZAP targeting and binding remains to be elucidated. This phenomenon may be further studied through the generation of different HIV-1 variants with increasing amounts of CpG sites in different regions of the genome, which could also be useful for identifying new factors that might be involved in the inhibition of HIV-1 replication.

## HIV-1 Codon Usage and Innate Immune Response

HIV-1-biased nucleotide composition can produce overstimulation of the type I interferon, suggesting that RNA sequences may be discriminated based on their nucleotide composition (Vabret et al., 2012). Type I interferon is a major antiviral cytokine that contributes to chronic immune system activation and progression to AIDS during HIV infection (Jacquelin et al., 2009). Pathogenicity is reportedly correlated with divergent nucleotide composition of HIV-1 compared to host (Vabret et al., 2012), suggesting that virus-host interactions might be altered by artificially changing the nucleotide frequency of the HIV-1 genome. With this aim, SIV codon usage was optimized to be closer to the average nucleotide composition of the SIV macaque host (Vabret et al., 2014). A synthetic SIV optimized with 169 synonymous mutations in *gag* and *pol* exhibited a 100-fold decrease of replicative capacity. Interestingly, this optimized variant also exhibited reduced ability to stimulate type I interferon in infected human and macaque PBMCs (Vabret et al., 2014). No reversion of the introduced mutations was observed after ten serial cell passages, suggesting that this variant may be a safe candidate for an attenuated vaccine. Still, further experiments should be performed to confirm the stability of this attenuated variant. Live attenuated SIV vaccines are highly protective in the macaque AIDS model (Koff et al., 2006). However, in addition to safety concerns, this optimized SIV variant raises intriguing questions related to the fact that a type I interferon response is necessary for a vaccine to shape adaptive immune responses and memory.

The Schlafen (SLFN) gene family was first discovered in 1998, as regulators of T-cell maturation. The name Schlafen means “to sleep” in German, and was chosen based on the observation that enhanced SLFN1 expression resulted in G_0_/G_1_ cell cycle arrest (Schwarz et al., 1998). SLFN genes are categorized as interferon-stimulated genes (ISGs), as their expression is induced by type I interferon. Some SLFN proteins possess RNA cleavage activity, and exhibit antiviral activity against RNA and DNA viruses. In particular, SLFN11 potently and selectively inhibits HIV-1 protein translation and virus production (Li et al., 2012), and is considered an HIV-1 restriction factor. SLFN11 also inhibits other retroviruses, including murine leukemia virus and feline immunodeficiency virus (Stabell et al., 2016), such that its antiretroviral effect has been classified as host-specific but virus-independent.

Interestingly, the inhibitory effect of SLFN11 on viral protein expression is intrinsic to the transcripts. SLFN11 acts at the point of virus protein synthesis by exploiting the unique viral codon bias toward A/U nucleotides, sequestering tRNAs in a codon-dependent manner (Figure 4). Accordingly, SLFN11 only affects WT HIV-1, and does not recognize synonymously recoded viruses in which the HIV-1 structural *gag* sequences are optimized for human codon usage (i.e., without A/U in the third position) (Li et al., 2012). Again, and similar to the findings obtained with SIV, HIV-1 replication was attenuated by codon optimization for host translation. This model is in line with findings that SLFN11’s antiviral activity extends to other viruses with rare codon bias (e.g., influenza, which also has a high A content) but not to adeno-associated virus or herpes simplex virus (Li et al., 2012). Overall, investigations in SIV and HIV-1 have shown that innate immunity restricts sequence landscapes by targeting specific sequences and sequence patterns that are primarily found in pathogens (Vabret et al., 2017).

## Conclusion

Synonymous rewriting of the HIV-1 genome is helping to elucidate essential genome functions. However, mammalian codon optimization is not straightforward, since synonymous mutations are often not neutral. On the other hand, intentional deoptimization of codon, codon-pair usage, or dinucleotide frequencies has been applied in several RNA virus genomes to generate new attenuated vaccines. Nevertheless, the safety and stability of these attenuated vaccines remain to be elucidated. Due to safety concerns, it is very difficult to envision the successful development of an attenuated HIV-1 vaccine. However, recoded HIV-1 variants can be used in gene therapy, as a vaccine vector for immunization against diverse microorganisms, or in immunotherapy to elicit specific innate immune responses to treat particular conditions. Importantly, artificial HIV-1 synonymous recoding has greatly increased our knowledge regarding its interaction with the host. Specifically, HIV-1 synonymous recoding impacts virus RNA nucleotide composition and RNA secondary structure which regulate splice-site use and viral gene expression. Together with structural RNA features, changes in nucleotide composition also affect HIV-1 susceptibility to endogenous cell innate responses and correlated with differences in clinical progression rates, suggesting a potential role of virus RNA nucleotide composition in HIV-1 *in vivo* pathogenicity.

## Author Contributions

AJ-P, SF, and MM discussed and wrote this review. All authors contributed to the article and approved the submitted version.

## Conflict of Interest

The authors declare that the research was conducted in the absence of any commercial or financial relationships that could be construed as a potential conflict of interest.
